# Supplementary material for: Development of a New Trapping System with Potential Implementation as a Tool for Mosquito-Borne Arbovirus Surveillance
Source: Insects. 2025 Jun 17;16(6):637. doi: 10.3390/insects16060637 (PMC12193242; doi:10.3390/insects16060637)
Supplement: Supplementary file 1 [file insects-16-00637-s001.zip › Supplementary file S1.pdf]

Development of a New Trapping System with Potential Implementation as a Tool for Mosquito-Borne Arbovirus Surveillance

Luísa Maria Inácio da Silva [1], Larissa Krokovsky [1,2], Rafaela Cassiano Matos [1,3], Gabriel da Luz Wallau [1,4,5,6] and Marcelo Henrique Santos Paiva [1,7,*]

1 Departamento de Entomologia, Instituto Aggeu Magalhães (IAM), Fundação Oswaldo Cruz (FIOCRUZ), Av. Professor Moraes Rego, s/n, Campus da UFPE, Cidade Universitária, Recife 50740-465, Brazil
2 Faculty of Mathematics & Science, Brock University, 1812 Sir Isaac Brock Way, St. Catharines, ON L2S 3A1, Canada
3 Programa de Pós-Graduação em Entomologia, Universidade Federal Rural de Pernambuco, Rua Dom Manoel de Medeiros, s/n–Dois Irmãos, Recife 52171-900, Brazil
4 Núcleo de Bioinformática (NBI), Instituto Aggeu Magalhães (IAM), Fundação Oswaldo Cruz (FIOCRUZ), Recife 50670-420, Brazil
5 Department of Arbovirology and Entomology, Bernhard Nocht Institute for Tropical Medicine, D-20359 Hamburg, Germany
6 Aggeu Magalhães Institute (IAM), Universidade Federal de Santa Maria (UFSM), Santa Maria 97105-900, Brazil
7 Núcleo de Ciências da Vida, Universidade Federal de Pernambuco (UFPE), Centro Acadêmico do Agreste-Rodovia BR-104, km 59-Nova Caruaru, Caruaru 55002-970, Brazil
* Correspondence: marcelo.paiva@fiocruz.br

Figure S1: Photography of BR-ArboTrap. A: BR-ArboTrap closed, showing the opening top lid. B: BR-ArboTrap opened, showing the oviposition recipient (not filled with water), and the two FTA systems.

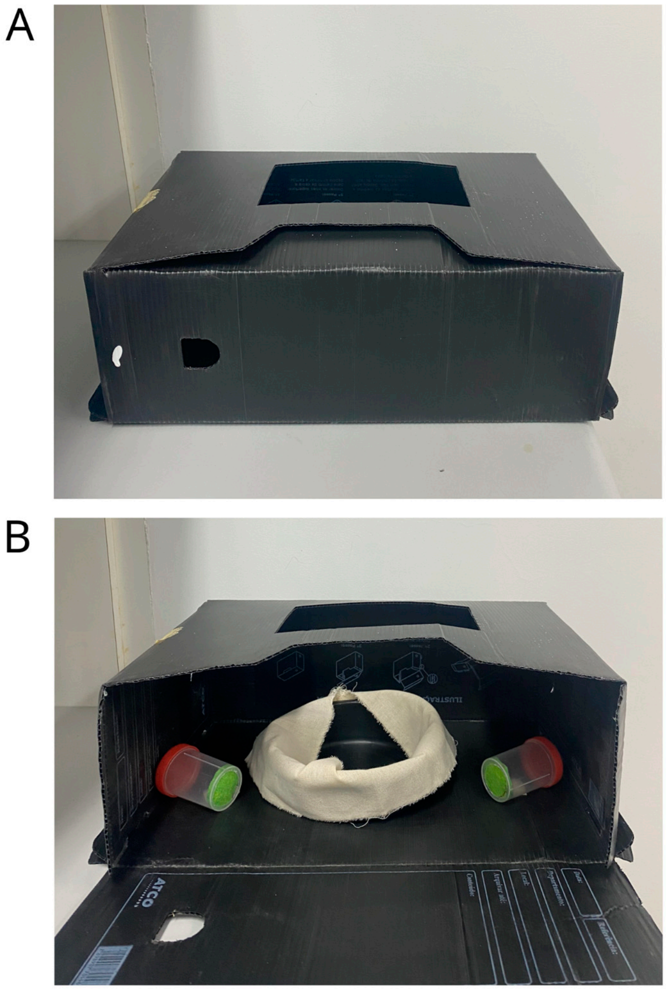

Table S1: Primers and probe sequences used in CHIKV detection.

Oligonucleotide Name	Sequence (5′–3′)	Amplicon Size (bp)	Reference
CHIKV 6856	TCACTCCCTGTTGGACTTGATAGA		(Lanciotti et al. 2007)
CHIKV 6981	TTGACGAGAGTTAGGAACATACC	126	
CHIKV 6919 P	VIC-AGGTACGCGCTTCAAGTTCGGCG-BHQ1		

Section S1: Description of standard curve synthese used in RT-qPCR assays.

This standard curve was generated following the method described by Kong et al. (2006), with modifications, as it follows [39]. To synthesize the standard curve, RNA was extracted from the CHIKV BRPE408 viral stock as previously described. cDNA was synthesized using the AMV Reverse Transcriptase System (Invitrogen™) in a final volume of 20 µL, according to the manufacturer's instructions. The resulting cDNA was used as a template in a PCR reaction using PCR Master Mix (Promega) and primers at a final concentration of 600 nM. The primers used were the CHIKV 6981 and the forward primer CHIKV 6856 modified with promoter T7 addition (5' TAATACGACTCACTATAGGGTCACTCCCTGTTGGACTTGATAGA'3). PCR conditions were as follows: initial denaturation at 95 °C for 15 minutes, followed by 35 cycles of 95 °C for 15 seconds, 55 °C for 15 seconds, and 72 °C for 30 seconds, with a final extension at 72 °C. This PCR product was then used to in vitro transcription with MEGAscript Kit® (Life Technologies). Subsequently, a DNase treatment was performed using the MEGAscript™ DNase (Thermo Fisher Scientific), according to the manufacturer's instructions. This was followed by a purification step following the instructions of MEGAscript Kit® to ensure the removal of residual contaminants. The amplified product was visualized on a 1.5% agarose gel stained with 0.1% ethidium bromide.

Table S2: RT-qPCR cycling conditions for CHIKV detection, based on the protocol described by Lanciotti et al. (2007).

Step	Temperature	Time	Repetition
1	45°C	15 min	
2	95°C	5 min	
3	95°C	5 seconds	45 X
4	60°C	45 seconds	

Figure S2: Amplification plot and standard curve of real-time RT-PCR for CHIKV detection. A-B: standard curve used in all experiments. C-D: from CHIKV-blood mixture used for artificial feeding. E-F: one plate from samples from experiments 1, 2, and 3;

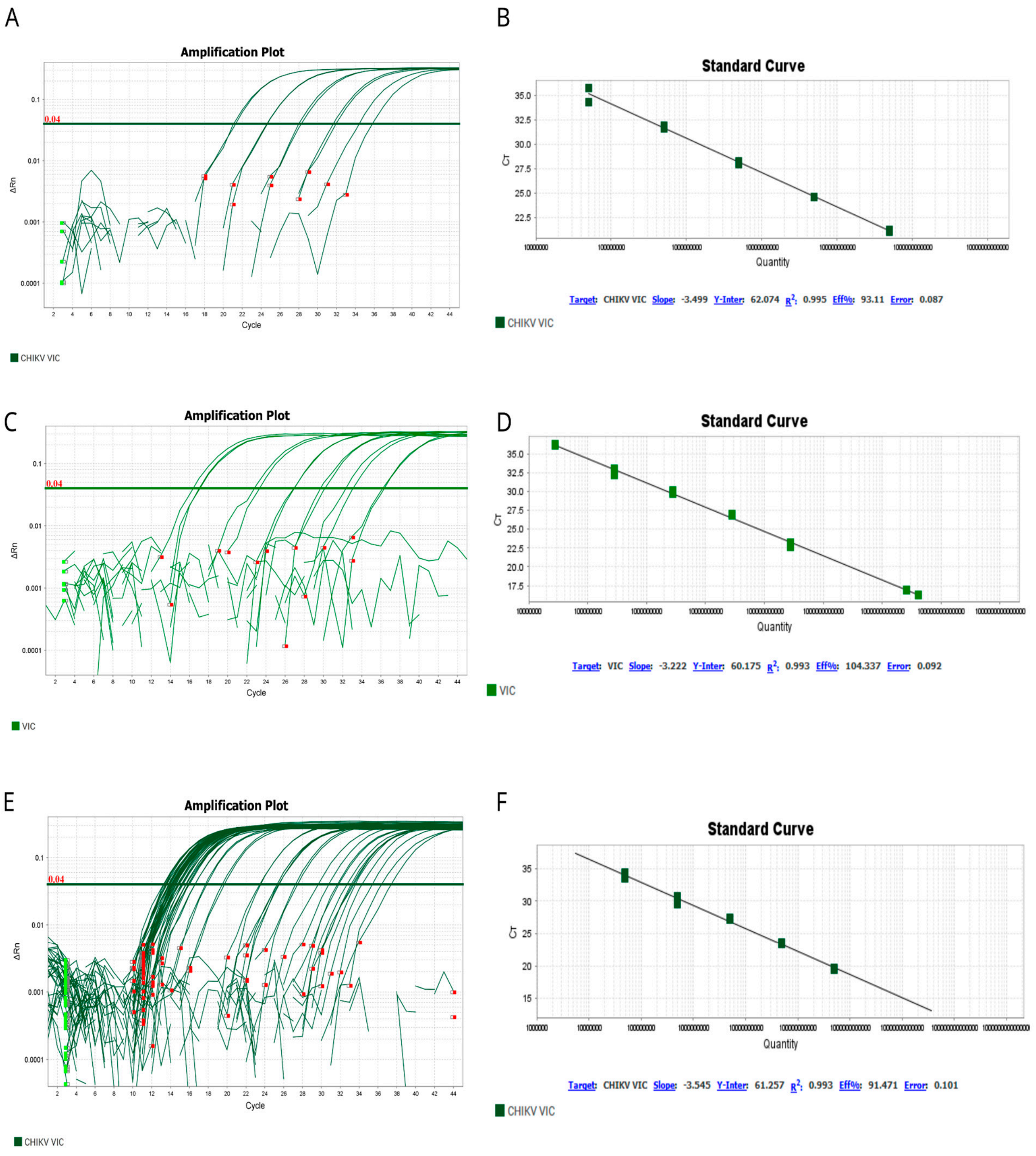

Table S3: Quantification of standard curve used for CHIKV in mosquitoes and FTA cards. Cq, Cycle quantification; SD, standard deviation.

Sample Name	Cq Mean	Cq SD	Quantity (RNA copies/mL)
CHIKV 10-2	21.138	0.102	499,200,000,000.000
CHIKV 10-3	24.614	0.033	49,920,000,000.000
CHIKV 10-4	28.122	0.157	4,992,000,000.000
CHIKV 10-5	31.781	0.223	499,200,000.000
CHIKV 10-6	35.049	1.034	49,920,000.000

Table S4: Parameters of standard curve used for quantification and detection of CHIKV in RT-qPCR assays.

Parameter	Value
Slope	-3.499
R2	0.995
eff%	93.11
Y-inter	62.074
