# Supplementary figures and images for: Development of a New Trapping System with Potential Implementation as a Tool for Mosquito-Borne Arbovirus Surveillance
Source: Insects. 2025 Jun 17;16(6):637. doi: 10.3390/insects16060637 (PMC12193242; doi:10.3390/insects16060637)

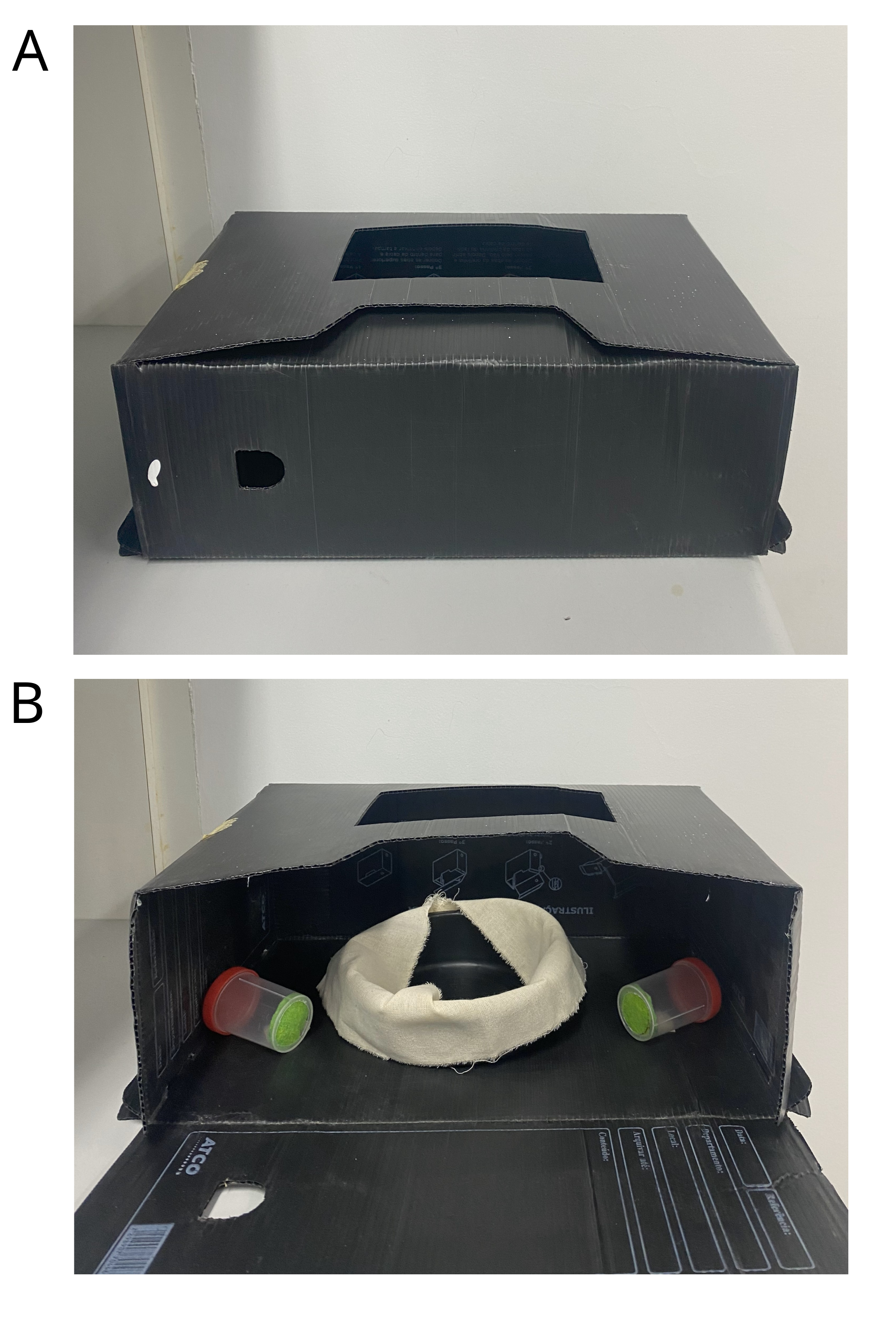

Supplement: Supplementary file 1 [file insects-16-00637-s001.zip › Supplementary figure S1.tiff]
